# Revue de la littérature sur l'impact du vieillissement démographique sur l'évolution des dépenses médicales: pays de l'Organisation de Coopération et de Développement Economiques et Maroc

**DOI:** 10.11604/pamj.2018.31.142.15248

**Published:** 2018-10-25

**Authors:** Imane Sninate, Ahmed Bennana

**Affiliations:** 1 Centre d'Etudes Doctorales Sciences de la Vie et de la Santé, Université Mohammed V Souissi, Faculté de Médecine et de Pharmacie, Rabat, Maroc

**Keywords:** Vieillissement démographique, consommation médicale, proximité du décès, progrès technologique, Demographic aging, medical consumption, proximity of death, technological progress

## Abstract

L'objectif de cette étude est de faire un point sur la littérature analysant la relation entre le vieillissement démographique et l'augmentation des dépenses médicales dans des pays où ce phénomène est déjà prégnant ainsi qu'au Maroc. Les 19 premiers articles les plus cités qui sont produits au cours de la période: 1995-2014, concernent les pays de l'OCDE et où l'impact du vieillissement sur les dépenses de santé est évalué quantitativement. En ce qui concerne le Maroc, la recherche a porté sur des publications émanant des instances gouvernementales nationales qui relatent l'évolution de la consommation médicale et son lien avec l'âge. Pour les pays de l'OCDE, les études qui incluent la proximité du décès dans les modèles d'explication des dépenses de santé, concluent que cette dernière explique mieux que l'âge la hausse des dépenses de santé. Quatre auteurs ont mis en évidence que les modèles d'explication des dépenses qui ne tiennent pas compte de l'approche du décès surestiment les résultats de projection. Dix études avancent que la hausse des dépenses de santé est imputable à des facteurs autres que le vieillissement démographique. Pour le Maroc, seul un rapport sur l'Assurance Maladie Obligatoire fait émerger un lien entre l'âge et les dépenses puisque la prévalence des maladies chroniques qui touchent davantage les personnes âgées occasionne des dépenses importantes à l'assurance maladie. Compte tenu de la vitesse du vieillissement au Maroc, il est nécessaire d'élaborer des études permettant la compréhension de la dynamique des dépenses de santé.

## Abstract (en)

This study aims to offer an overview of literature published on the relationship between population aging and healthcare expenditure growth in countries where this phenomenon is already significant and in Morocco. The first 19 most cited articles published over the period 1995-2014 are concerned with Organisation for Economic Co-operation and Development member countries as well as with countries where the impact of aging on healthcare expenditures has been evaluated quantitatively. With regard to Morocco, our research focused on publications from national governments regarding medical consumption growth and its link with age. Reasearch studies on OECD member countries that include proximity of death in explanatory models for healthcare expenditures, conclude that this last can explain better than age healthcare expenditure growth. Four authors highlight that explanatory models for healthcare expenditures which do not take into consideration proximity of death overestimate projection results. Ten studies suggest that healthcare expenditure growth is attributable to factors other than population aging. With regard to Morocco, only a report on compulsory health insurance shows that there is a relationship between age and expenditures since the prevalence of chronic diseases that affect mostly older people entails significant expenditures for compulsory health insurance. Given the speed of the aging in Morocco, it is necessary to conduct studies in order to understand the dynamics of healthcare expenditures.

## Introduction

Le vieillissement de la population défini comme l'augmentation du nombre des personnes âgées par rapport à la population totale, est la conséquence de la transition démographique qui consiste en le passage d'une population ayant des taux de mortalité et de natalité élevés à une population ayant des taux de mortalité et de natalité faibles, engendrant ainsi un allongement de l'espérance de vie et une baisse de la fécondité. Il est un phénomène planétaire majeur. Ainsi, la population mondiale âgée de 65 et plus représente 8,5% en 2015 et devrait atteindre 12% en 2030 et 16,7% en 2050.Toutefois, le degré et la vitesse du vieillissement varient d'un continent à un autre. Les continents les plus vieux sont l'Europe, l'Amérique du nord et l'Océanie où la part de la population ayant plus de 65 ans est respectivement de 17,4%, 15,1% et 12,5%. L'Asie concentre une population âgée de 7,9% mais connaîtrait une vitesse de vieillissement plus importante. L'Afrique qui abrite une population âgée de plus de 65 ans de 3,5%, reste, par contre un continent jeune en raison des niveaux élevés de fécondité, à l'exception des pays de l'Afrique du nord [1]. En ce qui concerne le Maroc, il est en train de connaître un processus de vieillissement rapide. Selon les statistiques du Haut-Commissariat au Plan, alors que les personnes âgées de 60 ans et plus (au nombre de 2,4 millions de personnes) ne représentaient en 2004 que 8 % de l'ensemble de la population marocaine, leur part dans la population totale devrait augmenter d'à peu près 5 points d'ici 2024 pour atteindre 13,1%. Six ans plus tard, en 2030, cette part serait de 15,4 % et la population atteindrait 5,8 millions d'individus [2]. En R39; 2042, un marocain sur cinq aurait plus de 60 ans. Ainsi, le Maroc aura connu en 18 ans, soit entre 2024 et 2042, un vieillissement comparable à celui qu'a connu la France métropolitaine en 80 ans, soit entre 1920 et 2000 [3]. Entre 1995 et 2015, la moyenne de l'espérance de vie des pays de l'OCDE a connu une augmentation annuelle moyenne de 0,3% passant de 75,1 ans à 80,3 ans au moment où l'espérance de vie à la naissance de la population marocaine a connu une hausse annuelle moyenne de 0,6% passant de 66,7 ans à 75,5 ans durant la même période [4]. Quant au taux de naissances qu'ont connu les pays de l'OCDE, il est passé, en moyenne, de 14,9 pour 1000 personnes en 1995 à 11,9 pour 1000 personnes en 2015, soit une baisse annuelle moyenne de 0,8%. Pour ce qui est du Maroc, ce taux est passé de 25,12 pour 1000 personnes en 1995 à 20,39 pour 1000 personnes en 2015, soit une diminution annuelle moyenne de 1% [5]. Certes, le taux des personnes âgées (personnes ayant plus de 65 ans) est plus élevé dans les pays de l'OCDE comparativement au Maroc: Il est de 14,13 % pour les Etats-Unis, 25,06% pour le Japon, Allemagne (21,27%), France (17,95%), Royaume-Uni (16,96%) et Italie (20,92%) au titre de 2013 [6] alors qu'elle n'est que de 6,1% au titre de 2014 au Maroc [2]. Mais les principaux facteurs déterminants dans le processus du vieillissement ont connu une évolution plus importante au Maroc par rapport aux pays de l'OCDE durant les vingt dernières années à savoir l'espérance de vie et le taux de naissances. Ainsi, le processus du vieillissement de la population marocaine qui n'est encore qu'à ses débuts connaîtrait une accélération dans les années à venir à laquelle les systèmes sociaux et de santé devraient être préparés en vue de relever les défis que la montée d'une population âgée risque de poser à l'avenir. Le vieillissement démographique est souvent associé à une hausse de la consommation médicale puisque l'avancement en âge entraîne souvent la détérioration de l'état de santé et mène au développement des maladies qui peuvent être chroniques. C'est ce qui explique que la question de l'impact du vieillissement sur les dépenses en soins de santé a été largement documentée notamment pour les pays occidentaux qui mettent la viabilité financière de leurs systèmes de sécurité sociale au centre de leurs préoccupations eu égard aux difficultés de financement auxquelles ils ont été confrontés au cours du vingtième siècle. Le présent article permet de faire un point sur la littérature analysant la relation entre l'augmentation de la population âgée et l'évolution des dépenses de la consommation médicale, d'une part, dans des pays où le phénomène du vieillissement démographique est déjà prégnant notamment les pays de l'Organisation de Coopération et de Développement Economique et, d'autre part, au Maroc.

## Méthodes

Nous nous sommes basés sur la bibliographie faisant l'objet du document " Vieillissement et santé: Eléments de bibliographie" -Mise à jour Juin 2015- [7] produit par l'Institut de Recherche et Documentation en Economie de la Santé que nous avons trouvé dans le cadre d'une première recherche exploratoire permettant d'identifier les idées forces de la littérature sur le lien entre le vieillissement et l'évolution des dépenses de santé. Puis une recherche a été effectuée par la combinaison des mots suivants *“ageing”* and “healthcare expenditures ” dans la base de données PubMed et dans quatre journaux de l'économie de la santé: *International Journal of Health Economics and Management, Journal of Health Economics, Health Affairs, The European Journal of Health Economics*. Cette recherche a débouché sur 111 articles, nous en avons sélectionné les 19 premiers articles les plus cités qui répondent aux critères suivants: la période de publication est comprise entre 1995 et 2014, concerne un des pays de l'OCDE et où l'impact du vieillissement sur les dépenses de santé est évalué quantitativement. Et nous avons exclu les articles qui émanent des organismes internationaux, se penchent uniquement sur les dépenses des maisons de vieillesse ou abordent les dépenses médicales et les dépenses des maisons de vieillesse sans que l'impact du vieillissement sur les dépenses médicales ne soit clair.

Pour la littérature concernant le Maroc: après avoir constaté l'absence d'articles scientifiques abordant l'impact du vieillissement sur les dépenses médicales au Maroc, nous avons procédé à une recherche des publications émanant des instances gouvernementales nationales qui relatent l'évolution de la consommation médicale et son lien avec l'âge.

## Etat actuel des connaissances

### Revue de la littérature pour les pays de l'Organisation de coopération et de développement économiques (OCDE)

Beaucoup d'études se sont penchées sur l'analyse de l'impact du vieillissement sur l'évolution des dépenses en soins de santé notamment dans les pays de l'OCDE. Certaines sont rétrospectives d'autres prospectives. Dans certaines études, l'impact du vieillissement est appréhendé comparativement à d'autres facteurs alors que d'autres études ont quantifié l'impact de l'âge à part. Dans la présente section, nous allons présenter les résultats de dix-neuf études.

### Proximité du décès

Zweifel et collègues qualifient le vieillissement d'un « redherring » ou un moyen de faire diversion. Ils ont publié en 1999 une étude économétrique sur la relation entre les dépenses de santé et l'âge menée sur les bases de données longitudinales présentant la consommation médicale sur les huit derniers trimestres de vie de personnes décédées pendant la période 1983-1992. Leurs résultats montrent que l'âge n'a aucun effet une fois le temps de la mort pris en considération pourvu que l'individu ait 65 ans et plus au moment du décès. Ainsi, la consommation médicale par habitant n'est pas affectée par le vieillissement de la population imputé à une augmentation de l'espérance de vie. Toutefois, la hausse de la population des personnes âgées semble déplacer la majeure partie des dépenses médicales à un âge plus élevé, ce qui fait que la dépense moyenne par habitant reste inchangée [8]. Ce constat concorde avec les conclusions de Lubitz et collègues (2003) qui ont étudié la relation entre l'état de santé et l'espérance de vie à l'âge de 70 ans ainsi que les dépenses médicales cumulatives à partir de l'âge de 70 ans jusqu'à la mort en se basant sur les données qui proviennent de l'enquête sur les bénéficiaires actuels de Medicare (MCBS) couvrant la période 1992-1998. Ils ont conclu que les personnes âgées en meilleure santé avaient une espérance de vie plus longue que celles qui avaient un état de santé dégradé, mais avaient des dépenses médicales cumulatives similaires jusqu'à la mort: une personne sans limitation fonctionnelle à l'âge de 70 ans avait une espérance de vie de 14,3 ans et des dépenses cumulatives prévues d'environ 136 000 $ et une personne ayant une limitation dans au moins une activité de la vie quotidienne avait une espérance de vie de 11,6 ans et des dépenses cumulatives prévues d'environ 145 000 $ [9]. Seshamani et Gray (2004a) reproduisent la méthodologie de Zweifel et collaborateurs pour les données d'Oxfordshire (Angleterre), durant 1970-1999 en incluant d'autres variables pour expliquer la consommation médicale. Leurs résultats suggèrent que la proximité de la mort est fortement liée aux coûts hospitaliers dès 15 ans avant la mort et que cet effet est resté constant les trois dernières décennies [10]. Dans une étude ultérieure, Seshamani et Gray (2004b), utilisant la même base de données, constituent des cohortes d'âge correspondant à la population âgée de 65, 75, 85 et 95, respectivement, et estiment un modèle amélioré qui analyse les dépenses de santé engagées par ces patients dans les 24 années précédant leur décès. Leurs résultats soulignent le manque d'importance de l'âge et du sexe, avec des coefficients de 0,027 et 0,014, respectivement, tandis que les coefficients des variables se rapportant aux 15 ans précédant le décès affichent une tendance à la hausse, jusqu'à une valeur de 0,571 pour l'année juste avant la mort [11]. D'ailleurs, le fait que le décès survient souvent suite à un déclin de l'état de santé qui engendre un recours aux soins et une consommation médicale intenses explique que l'approche de la mort soit un meilleur prédicteur des dépenses de santé. Lubitz et Spillman (2000) ont conclu que plus l'âge du décès augmente, les dépenses de fin de vie baissent: les dépenses de Medicare au cours des deux dernières années de vie sont de 37 000 $ pour les personnes qui meurent à l'âge de 75 ans et 21 000 $ pour ceux qui meurent à l'âge de 95 ans [12].

(Brockmann , 2000) a démontré également que ce n'est pas l'âge mais la proximité du décès qui est déterminante dans les coûts des soins de santé qui baissent avec l'âge en Allemagne et suggère que les soins de santé sont informellement rationnés en fonction de l'âge et du sexe du patient, chose qui pourrait être imputée -en ce qui concerne l'âge- à la décision professionnelle du médecin de ne pas prodiguer certains soins et interventions car un décès qui survient pendant le traitement peut être considéré comme un échec médical. En outre, il existe moins de connaissances sur le traitement des patients plus âgés car ces derniers ont été largement exclus des essais cliniques afin de minimiser les problèmes analytiques causés par la comorbidité [13]. (Yang, Norton et Stearns, 2003) ont mené des analyses graphiques et descriptives sur la base de données issues de L'Enquête sur les bénéficiaires actuels de Medicare (MCBS) pour les années 1992-1998. Leurs résultats ont montré, entre autres, que la proximité du décès est la plus importante raison de l'augmentation des dépenses de l'hospitalisation qui baissent à mesure que l'âge du décès augmente [14]. Hansen et collègues (2002) ont mené une étude de projection des coûts futurs des soins hospitaliers et des services de soins de santé primaires durant la période 1995-2020 au Danemark sur la base de changements démographiques, avec et sans prise en compte des coûts élevés de la dernière année de vie. Ils ont constaté que les coûts des soins de santé devraient augmenter de 18,5% selon la méthode de projection traditionnelle et de 15,1% selon la méthode améliorée tenant compte des coûts de la dernière année de vie. Ces résultats suggèrent que le vieillissement en soi semble avoir un impact considérable sur les coûts futurs des soins de santé et que les coûts élevés de la dernière année de vie devraient être pris en compte dans les projections des coûts futurs des soins de santé [15]. (Hakkinen et collaborateurs, 2008) ont mené des prédictions des dépenses de santé ainsi que des soins dispensés au niveau des maisons des soins infirmiers sur la base de données finlandaises en incluant les survivants et les décédés dans les analyses et ont comparé également les résultats d'un modèle qui tient compte de la proximité de la mort avec ceux d'un modèle “naïf”, qui ne comprend que l'âge , le genre et leurs interactions .Ils ont constaté, pour les personnes qui ne bénéficient que de soins de santé, une relation positive claire entre les dépenses et l'âge uniquement pour le centre de santé et les soins psychiatriques pour patients hospitalisés et que cette relation est atténuée lorsque la proximité de la mort est prise en compte. Quant à la projection totale à l'horizon 2036 des dépenses y compris celles des maisons des soins infirmiers, l'approche qui prend en compte la proximité de la mort donne une estimation de 13% plus faible par rapport à l'approche naïve [16]. Ceci s'explique par le fait que si, d'une part, le niveau élevé des dépenses de santé dans les âges plus avancés est fortement dépendant du coût élevé des soins en fin de vie et que, d'autre part, l'espérance de vie s'accroît significativement au cours de la période analysée, la baisse de la mortalité par âge va faire diminuer le niveau moyen des dépenses de santé par âge. (Gray, 2005), dans son papier où il a retracé l'évolution de la recherche par rapport à la question de l'impact du vieillissement sur les dépenses de santé a cité également, que « la croyance largement répandue selon laquelle il existe une relation mécaniste entre une population vieillissante et la croissance annuelle de la demande de soins de santé et des dépenses nationales de santé est inexacte » et que la recherche qu'il a effectué « montre également de façon concluante que le temps de mort est un prédicteur nettement meilleur que l'âge des dépenses de santé, et que lorsque cela est incorporé dans les projections des dépenses de santé futures, les taux de croissance prévus sont généralement abaissés» [17].

### La hausse des dépenses de santé est due, aussi, à d'autres facteurs que le vieillissement qui peuvent être plus importants

(Gray, 2005) a également ressorti de sa revue de littérature que « les changements dans la structure démographique et dans l'état de santé ne sont qu'une partie d'un ensemble beaucoup plus large d'influences sur les dépenses de santé futures» [17]. Breyer et Felder (2006) ont appliqué aux projections de la population allemande entre 2002 et 2050 les profils de dépenses par âge pour les deux sexes-obtenus à partir de données suisses-, séparément pour les personnes dans leurs quatre dernières années de vie et pour les survivants. Ils ont conclu qu'avec l'application d'une approche de projection distinguant les survivants et les décédés, la dépense moyenne de santé par habitant passerait de 2 696€ en 2002 à 2 959 euros sans prise en compte de l'amélioration de l'espérance de vie- et 3 102 euros-en cas de prise en compte de l'amélioration de l'espérance de vie en 2050, alors que seule la structure par âge de la population change et tout le reste demeure constant au niveau actuel, et à 5 485€ avec une augmentation des coûts exogènes liés à la technologie médicale de 1% par an. Une projection “naïve” basée uniquement sur la répartition par âge des dépenses de soins de santé, mais sans distinction entre survivants et décédés, donne une valeur de la dépense moyenne par habitant de 3 217€ si on ne tient pas compte de l'effet de la technologie et 5 688€ si l'évolution de la technologie est prise en considération pour 2050. Ainsi, l'erreur consistant à exclure l'effet « coûts de la mort » est faible par rapport à l'erreur de sous-estimation des conséquences financières de l'expansion de la technologie médicale [18]. Dans son étude de l'impact des changements démographiques probables sur les dépenses de Medicare pour les personnes âgées, Cutler (1998), s'est appuyé sur l'évolution des dépenses ainsi que sur les résultats des recherches antérieures et a conclu que les changements dans les tendances du handicap et de la mortalité devraient réduire les dépenses médicales pour les personnes âgées. Ainsi, l'allongement de l'espérance de vie fait que plus de personnes âgées vont mourir à un âge plus avancé, et les coûts de fin de vie diminuent habituellement avec l'âge au décès. De plus, les taux d'invalidité baissent chez les survivants. Ces deux facteurs contribueraient à la réduction des dépenses médicales. Cependant, la dépense médicale moyenne à état de santé donné va connaître une hausse en raison des progrès médicaux [19]. Fuchs (1999) a discuté le rôle important de la technologie dans la croissance des dépenses de santé aux Etats-Unis. Il avance que l'apparition d'un nouveau médicament ou d'une nouvelle technique de diagnostic a un effet modeste sur les dépenses de santé au départ mais son développement, son raffinement et sa diffusion entraînent une augmentation importante des dépenses [20].

Reinhardt (2003), dans une revue des différentes études menées aux États-Unis qui ont évalué l'impact du vieillissement sur les dépenses de santé, constate que la diffusion des technologies médicales, l'augmentation du revenu par habitant, l'asymétrie du pouvoir de marché des soins de santé qui donne à l'offre une influence considérable sur la demande et la pénurie de professionnels de la santé sont les véritables moteurs de la croissance des dépenses américaines de santé alors que le vieillissement est un processus trop lent pour être classé comme principal facteur de la hausse des dépenses de santé [21]. Dormont et collaborateurs (2006) ont mené une étude sur des échantillons représentatifs de de la population française observés respectivement en 1992 et 2000 afin d'évaluer la contribution des effets suivants: changements démographiques, changements dus à l'évolution de morbidité (dégradation ou amélioration de l'Etat de santé) et changements dans les pratiques des médecins pour une morbidité donnée dans la croissance des dépenses de soins de santé. Ils ont ressorti que l'augmentation des dépenses en soins de santé en raison des changements démographiques a été très faible par rapport aux effets des changements dans les pratiques: Alors que les dépenses totales ont connu une augmentation de 53,9%, l'impact des changements dans les pratiques a été estimé à 12,9% - soit 3,8 fois la hausse des dépenses de soins de santé imputée à des changements dans la structure par âge de la population estimée à 3,4% [22]. Matteo (2005) a examiné les déterminants des dépenses réelles de santé par habitant afin d'évaluer l'impact du revenu, de la répartition par âge et du temps au Canada (1975-2000) et aux États-Unis (1980-1998) estimant deux modèles pour chaque pays. Il a constaté que les dépenses réelles de santé par habitant sont positivement et significativement liées aux variables sus-visées et qu'il existe des différences importantes dans l'impact de ces variables sur les dépenses en fonction de la complexité de la spécification de la variable choisie et des modèles estimés: des modèles simples de dépenses de santé constatent que la croissance de la proportion de la population âgée de 65 ans et plus est responsable d'une grande partie de l'augmentation des dépenses de santé alors que les modèles qui utilisent une spécification plus complexe pour l'âge ainsi que les modèles qui tiennent compte des variables de l'indicateur du temps trouvent que l'âge est de loin la variable la plus importante. Les effets du temps peuvent être interprétés comme une variable proxy des changements technologiques comme elles peuvent englober également les effets des changements de politique, des variables omises, des changements dans les préférences et les attentes des consommateurs en matière de santé et peuvent expliquer environ les deux tiers de l'augmentation des dépenses de santé réelles par habitant [23]. Dans une analyse comparative des tendances du vieillissement entre huit pays industrialisés à savoir l'Australie, le Canada , la France, l'Allemagne, le Japon, la Nouvelle Zélande, les Etats unis et le Royaume Uni où la part des dépenses de santé consacrées aux personnes âgées varient entre 1/3 et 1/2 des dépenses totales entre 1993 et 1995, Anderson et Hussey (2000) ont noté qu'il y a une très faible corrélation (-0,07) entre la part des dépenses des personnes ayant plus de 65 ans dans le Produit Intérieur Brut dans les pays sus-visés et la part de cette catégorie de la population par rapport à la population totale suggérant ainsi qu'il y a d'autres facteurs plus importants prédicteurs de la dépense de santé [24]. José Martin et collègues 2013, dans leur revue de la littérature sur les déterminants de la consommation médicale portant sur 20 articles, avancent « *qu'il n'existe pas de preuve empirique solide appuyant le fait que le vieillissement de la population est l'un des principaux déterminants des dépenses de santé*» et jugent que des facteurs tels que le progrès technologique, la proximité du décès et la décentralisation territoriale des soins de santé doivent être inclus dans les modèles d'explication des dépenses médicales [25]. (Pammolli et collaborateurs, 2012), ont étudié la contribution du revenu, du vieillissement démographique , du progrès technologique, de la participation des femmes au travail et des variables budgétaires publiques dans la dynamique des dépenses médicales de 15 pays européens et ont conclu qu'en plus du Produit Intérieur Brut, le vieillissement et la participation des femmes au travail contribuent positivement à la croissance des dépenses totales de santé .De plus , le fait que la part de la population ayant plus de 65 ans soit corrélée positivement avec les dépenses publiques et négativement avec les dépenses privées est susceptible d'aggraver les problèmes de financement dans le futur [26].

### Maroc

En raison de l'absence d'articles scientifiques abordant l'impact du vieillissement sur les dépenses médicales au Maroc, nous avons procédé à une recherche des publications et rapports émanant des instances gouvernementales nationales qui relatent l'évolution de la consommation médicale par tranches d'âges, notre recherche a débouché sur le Rapport Annuel Global de l'Assurance Maladie Obligatoire (AMO) produit par l'Agence Nationale de l'Assurance Maladie [27]. Ce rapport retrace l'évolution des indicateurs démographiques, de recettes et de dépenses pour les deux principaux régimes de l'AMO des salariés des secteurs public et privé gérés respectivement par la Caisse Nationale des Organismes de Prévoyance Sociale (CNOPS) et la Caisse Nationale de la Sécurité Sociale (CNSS) et qui couvrent 26% de la population marocaine. Il en ressort que la part de la population âgée de plus de 60 ans couverte a connu une évolution annuelle moyenne de 2,5% passant de 15,2% en 2012 à 16,4% en 2015 pour la CNOPS et de 1,9% pour la CNSS passant de 8,4% en 2012 à 8,9% en 2015. De plus, cette catégorie de population a un recours aux soins plus important que les personnes ayant moins de 60 ans: Pour la CNSS, le taux de recours aux soins pour les personnes ayant plus de 60 ans est de 42% contre 16% pour les moins de 60 ans. Quant à la CNOPS, ce taux est de 52% pour les personnes ayant plus de 60 ans contre 45% pour les personnes ayant moins de 60 ans. Cet écart est encore plus prononcé pour les personnes touchées par au moins une maladie chronique: Le taux des personnes touchées par une maladie chronique et qui recourent aux soins est de 12,8% pour la CNOPS et 14,9% pour la CNSS pour les personnes ayant plus de 60 ans contre 2,8% et 1% pour les personnes ayant moins de 60 ans respectivement pour la CNOPS et la CNSS. Eu égard aux coûts de traitement élevés des maladies chroniques, ces dernières représentent une charge financière importante pour les caisses d'assurance maladie car la population totale touchée par au moins une maladie chronique concerne 2,8% de la population totale et s'approprie 48,2% des dépenses totales au titre de 2015. De plus, la population touchée par au moins une maladie chronique a connu une évolution annuelle moyenne de 11,1% entre 2010 et 2015.

## Conclusion

Comme on le constate dans cette revue de la littérature, si l'allongement de l'espérance de vie fait penser que le vieillissement est un facteur important dans la hausse des dépenses de santé puisque plus de personnes âgées seront dans les groupes d'âge plus avancés où les coûts médicaux augmentent , il s'avère que son impact est moins intense en raison de l'effet de deux autres facteurs: Tout d'abord, l'augmentation de l'espérance de vie signifie qu'une part plus faible des personnes âgées sera dans la dernière année de vie où les coûts médicaux sont généralement très élevés. En outre, plus de personnes âgées vont mourir à un âge plus avancé, et les coûts de fin de vie diminuent habituellement avec l'âge au décès. Le progrès de la médecine qui stimule le recours aux soins, puisqu'il permet de dépister des maladies qui ne pouvaient pas être détectées et de traiter d'autres qui étaient auparavant incurables, entraîne la modification des pratiques des médecins et déclenche une demande latente des patients joue également un rôle important dans la hausse des dépenses de santé. En ce qui concerne le MAROC, il est nécessaire de mener des travaux de recherche afin de mettre en évidence la relation entre l'évolution des dépenses de santé et le vieillissement démographique et autres facteurs en vue de mieux comprendre la dynamique des dépenses, de mettre à la disposition des planificateurs et décideurs du système de santé des éléments probants qui leur permettraient d'agir en vue d'améliorer la performance du système.

### Etat des connaissances actuelles sur le sujet

- L'impact du vieillissement démographique sur les dépenses médicales est moins intense si on tient compte de la proximité du décès dans l'explication des dépenses.

### Contribution de notre étude à la connaissance

- L'absence d'articles scientifiques sur l'impact du vieillissement démographique sur l'augmentation des dépenses de santé au Maroc;
- Eu égard à la vitesse du vieillissement démographique au Maroc, il est nécessaire d'élaborer des études en vue de mettre en évidence la contribution de chaque facteur (vieillissement démographique, progrès technologique, revenu, ..) dans l'explication de la dynamique des dépenses de santé et de constituer des bases de données au niveau des caisses d'assurance maladie et des hôpitaux contenant l'information indispensable à la réalisation des études sus-visées notamment l'âge du décès;
- De plus, et au vu des chiffres relatifs au contexte marocain qui montrent que la poursuite de la tendance du vieillissement serait accompagnée d'une augmentation de la prévalence des maladies chroniques, il s'avère judicieux d'une part d'introduire cette variable dans la modélisation quantitative des dépenses médicales et d'autre part d'identifier puis d'analyser également les facteurs déterminants dans l'évolution des dépenses des personnes atteintes de ces maladies coûteuses, à part l'âge.

## Conflits d'intérêts

Les auteurs ne déclarent aucun conflit d'intérêts.
